# Accurate prediction of gaseous isotopes Xe and Kr concentrations from the burnup of nuclear fuel using simple regression algorithm

**DOI:** 10.1371/journal.pone.0288329

**Published:** 2023-07-13

**Authors:** Sherif S. Nafee, Amir M. Al-Ramady, Nader M. Mohamed, Manal F. Alshammari

**Affiliations:** 1 Physics Department, Faculty of Science, King Abdulaziz University, Jeddah, K.S.A; 2 Physics Department, Faculty of Science, Alexandria University, Alexandria, Egypt; 3 Department of Nuclear Engineering, Faculty of Engineering, King Abdulaziz University, Jeddah, K.S.A; 4 Reactors Department, Nuclear Research Center, Egyptian Atomic Energy Authority, Cairo, Egypt; 5 Physics Department, Faculty of Science, University of Hail, Hail, K.S.A; Damascus University, SYRIAN ARAB REPUBLIC

## Abstract

Mathematical techniques for modeling and simulating dangerous or complex systems, such as nuclear technology systems, often require high-performance computing to process and analyze available data. In this paper, simple and quick method to support studies and research related to nuclear fuel is presented. This reasonably simple method helps to predict different concentrations of actinides and fission products in nuclear fuels without the need for expensive specialized programs and highly-trained researchers. The great importance of this approach is the speed of predicting the components of nuclear fuel concentrations, which in turn leads to quick decision-making, such as the possibility of operating fuel at higher burnup values, predicting the amount of gases resulting from nuclear fission (which may accumulate and cause problems in nuclear fuel such as volume swells), and other important decisions in nuclear fuel technology. The predicted equations have been generalized for higher values of burnup and compared with comparable results from MCNP codes. The equations deduced in calculating the different concentrations of xenon and krypton isotopes resulting from fission in burnup of nuclear fuel showed very precise results with discrepancies (magnitude of an error between the data points and the corresponding predicted ones) less than 2%. The suggested method offers a great advantage for researchers, which are the use one of any simple or common computational programs available to most researchers and do not need much experience to deal with, such as MATLAB, Excel that are easy to use for regression analyses. In this paper, the advantages of the proposed method are explained along with the limitations of its use.

## Introduction

Development of the safe management of nuclear fuel and nuclear waste requires comprehensive predictions of the compositions of actinides and fission products using computational codes. These predictions help researchers develop appropriate strategies to reduce radiation exposure and environmental impact during all phases of nuclear fuel handling. In spent fuel, the compositions of both actinides and fission products depend on three factors: the initial fuel composition, fission yields of fissionable actinides present in the fuel, and the burn-up of the fuel [1].

One of the most important data needed in dealing with spent fuel strategies is to know the chronological evolution of nuclear fuel components. It breaks down into lanthanides and actinides suspended in the fuel itself, metallic fission products, some oxides, and fission produced gases. These gases are finely dispersed bubbles within the fuel matrix. These bubbles can build up in the nuclear fuel over time, causing volume swells, which in turn may cause a breach in the fuel cladding that surrounds the fuel. This cladding penetration is highly detrimental to the long-term behavior of the fuel in storage, which may increase corrosion and release of radionuclides into the surrounding environment [2].

The behavior of gaseous fission products greatly affects nuclear fuel performance while the reactor is in operation or even after shutdown. Many publications have shown that most of these bubbles are a group of non-reactive fission gases (xenon and krypton) emitted during the operation or shutdown of the reactor and are related to the level of burnup. Therefore, researchers have been interested in studying the growth of these gas bubbles resulting from fission within different types of nuclear fuel [3–7].

The fission product concentrations (Xe, Kr) are important in studying both the release and swelling effect of fission gases in nuclear fuel. Moreover, the volatile fission product concentrations are also necessary [8, 9]. Fission products decay and fission processes are the two sources of the gaseous fission products. As noted, xenon and krypton represent most of the fission gases; each is produced as single atoms inside the nuclear fuel, which then gather over time until gas bubbles are formed. Research on fissile gases has very much focused on those gaseous isotopes with long half-lives [10].

Some stable isotopes of xenon and krypton were discovered by mass spectrometry between 1920 and 1922, such as; Xe-129, 130, 131, 132, 134 and 136 and Kr-82, 83, 84, 86. Whereas, Xe-133 and 135 of half-lives (125.8 and 9.14 hours) were discovered by light particle reactions. In addition to Kr-85 of half-life 339430000 Seconds, which was discovered in 1940 by neutron capture reactions [11, 12].

The modeling of nuclear fuel performance using specialized computer codes is important for design and for safety analysis. These codes vary with the type of fuel used. In nuclear fuel technology, there are many models of nuclear fuel and fuel rod performance codes developed around the world. In general, there are some codes that simulate normal operating behavior and others that simulate behavior under off-normal conditions. One of the major difficulties with nuclear fuel performance modeling is that it is done with dedicated computer codes, which require extensive knowledge of modeling. It also requires computing devices with high processing power in addition to the need for licenses to run [13].

Our research is characterized by deducing fuel composition of nuclides in a way that does not depend on complex spent fuel codes, and does not depend on spent fuel databases, which are often incomplete. In this paper, we have used a simplified approach to predict gaseous isotopic inventories (the amount of nuclear material present) of both xenon and krypton for nuclear fuels greater than a certain limit of burnup. This predictive work contributes to addressing the problem of the lack of specific data in nuclear fuel technology. This research also may help to make quick decisions about how long to shut down nuclear reactors after a certain limit of burn by predicting the amount of poison isotopes, such as Xe-135, which is the most powerful known neutron absorber. Also, this research could be useful in supporting recent trends in enabling nuclear reactors to operate up to very high burnup (around 100 GWd/tHM).

## Methodology

All regression analyses were performed using MATLAB R2022A software, which provides an excellent platform for regression and fitting using matrix operations [14]. Radioisotope inventories was done by MCNPX 2.7.0. computational code [15]. The pins of a 17x17 Westinghouse pressurized water reactor fuel assembly lattice cells were simulated as shown in “S1 Fig”. A criticality calculation with BURN cards was used to calculate the fuel burnup and inventories for time intervals. The neutron cross section data were extracted from the ENDF/B-VII.0. data library. Fuel enrichment was considered for fuel enrichment of 4.5 w/o for the current practice of a 51 GWd/tHM burnup.

Results from MCNP are divided into two parts. The first, up to 25 GWd/tHM burnup, is used in the regression process using the least squares method [16–18] to derive the equations for the concentrations of fission product Xe and Kr. The second part of the results, up to 51 GWd/tHM burnup, is compared with the derived equations to ensure accuracy by calculating the discrepancies between them to consider whether these equations were accurate predictors of the fission product concentration formulations.

The least square regression method shows a major disadvantage since it is very sensitive to the selection of starting values [19]. Therefore, we neglected the first point in each case, which represents zero Burnup. In addition, it has potential limitations, such as its outliers. Although we can neglect the outliers if they do not have a significant effect on the fit of the model leading to incorrect conclusions [20], all data points (MCNP output) did not have any outliers, and this is evident when we plot the concentration of any required fission product with the Burnup.

To verify the relevance of the results derived from the equations (*Y*_*Eq*_), discrepancies were calculated using these inferred data and those calculated using the MCNP (*Y*_*i*_) for each nuclide according to the following equation:

$$\Delta \%\;=\;\frac{Y_{Eq}\;-\;Y_{i}}{Y_{i}}\;*\;100$$ (1)

For each desired fission-product isotope, a polynomial regression was made by plotting “Concentration” (g/tHM) of the fission-product isotope as the y-variable versus “Burnup” (GWd/tHM) as the independent x-variable. A simple MATLAB code was written to make a polynomial regression and the statistical indicators for every regression, ensuring that the model fits the data or the goodness of regression. These statistical indicators are root mean squared error (RMSE) as shown in Eq (2) and R-squared (R^2^) as shown in Eq (3). RMSE tells us the typical error between the *Y*_*Eq*_ value made by the regression code and *Y*_*i*_ value. In addition, R^2^ tells us how well the *Y*_*Equation*_ can explain the variation in the response variable.

$$RMSE\;=\;\sqrt{\frac{1}{N}\;\sum_{1}^{N}{\left(Y_{Eq}\;-\;Y_{i}\right)}^{2}\;}$$ (2)

$$R^{2}\;=\;1\;-\;\frac{N*MSE}{\sum_{1}^{N}{\left(Y_{i}-\bar{Y}\right)}^{2}}$$ (3)

where N is the number of MCNP data points, MSE = (*RMSE*)^2^, and $\bar{Y}\;=\;\sum_{1}^{N}Y_{i}$.

For each studied isotope, regressions of first, second and third degrees were done, then a comparison was made between the statistical indicators for each of them so that the best solution could be chosen according to the following criteria:

1. The higher the R^2^ value, the better a model fits a dataset (0 ≤ R^2^ ≤ 1).
2. The lower the RMSE, the better a model fits a dataset.

It was initially assumed that the relationship between variables x (time) and y (concentration) is a polynomial of degree n, a polynomial regression. Then the main equation would be Eq (4) and its solution is found by deducing the constants *C*_*i*_.


$$Y_{Eq}(t)\;=\;P_{n}(t)\;=\;C_{0}\;+\;C_{1}\;*\;t\;+\;C_{2}\;*\;t^{2}\;+\;C_{3}\;*\;t^{3}\;+\;\cdots \;+\;C_{n}\;*\;t^{n}$$ (4)

The reason we started with a polynomial regression for measured data that depends on a controlled variable is because it is the most popular function in data analysis. This was confirmed after obtaining the results, as it was found that this method was suitable for all cases and, indeed, most of the results reached a high degree of accuracy.

To start a least squares polynomial regression, it is first checked that n is greater than N; we then define:

$$P_{n}\left(t_{i}\right)\;=\;Y_{i},\;i\;=\;0,\;1,\;2,\;\ldots ,\;N$$ (5)

To minimize the mean square error, C_i_ is chosen so that $f(C)\;=\;\sum_{i=0}^{i=N-1}{\left(P_{n}\left(t_{i}\right)-\;Y_{i}\right)}^{2}$ is a minimum. To achieve this, its differential must be equal to zero:

$$\frac{\partial f(C)}{\partial C_{i}}\;=\;0,\;i\;=\;0,\;1,\;2,\;\ldots ,\;n$$ (6)

For example, in the case of a 2^nd^ order polynomial regression (n = 2), we can differentiate Eq (6) with respect to C_0_, C_1_ and C_2_ and this gives the following three equations:

$$\sum_{i=0}^{N-1}\;C_{0}\;+\;C_{1}\;*\;t_{i}\;+\;C_{2}\;*\;t_{i}^{2}\;-\;Y_{i}\;=\;0$$ (7)

$$\sum_{i=0}^{N-1}\left(C_{0}\;+\;C_{1}\;*\;t_{i}\;+\;C_{2}\;*\;t_{i}^{2}\;-\;Y_{i}\right)*t_{i}\;=\;0$$ (8)

$$\sum_{i=0}^{N-1}\left(C_{0}\;+\;C_{1}\;*\;\;t_{i}\;+\;C_{2}\;*\;t_{i}^{2}\;-\;Y_{i}\right)\;*\;t_{i}^{2}\;=\;0$$ (9)

Therefore, by solving these three “equations for quadratic regression”, constants C_0_, C_1_ and C_2_ are found and Eq (4) is solved. Similarly, Eq (4) can be solved in the case of regression from other degrees.

## Results and discussion

The very simple flowchart in “S2 Fig” illustrates the sequence of calculations followed. It gives a brief description of the method used to predict the concentrations of isotopes of xenon and krypton from zero to 51 GWd/tHM burnup, showing all the inputs and outputs of both the MCNP and MATLAB programs, and clarifies the mathematical and statistical operations used in this method.

The burnup of our fuel produced 9 isotopes of xenon: Xe-128, Xe-129, Xe-130, Xe-131, Xe-132, Xe-133, Xe-134, Xe-135, and Xe-136 and 5 isotopes of krypton: Kr-82, Kr-83, Kr-84, Kr-85, and Kr-86.

The isotopic inventory (concentration against burnup) data for the Xe and Kr isotopes shown in “Table 1” for burnup less than 25 GWd/tHM were taken from the output file of MCNP. This output includes actinides and both light and heavy fission products. For each isotope, first, second and third-degree polynomial regression analyses were performed, then statistical indicators were calculated for each case and the best fitting equation was chosen to represent each case. Each time Eq (4) was solved and values of the constants were obtained.

**Table 1:** Parameters of Eq (4): f(x) = p1*x3 + p2*x2 + p3*x + p4.

					Goodness of fit		
Isotope	P1	P2	P3	P4	SSE	R-Square	RMSE
Xe-128	0.00002227	0.00105	-0.001529	0.001491	3.231E-06	1	0.0007338
Xe-129	6.451E-07	-0.00002336	0.0004127	-0.002544	7.793E-09	1	0.00003948
Xe-130	0.00004196	0.00229	0.002646	-0.003606	0.00002481	1	0.001883
Xe-131	0	-0.09592	17.15	-7.827	0.8625	1	0.351
Xe-132	0	0.1885	25.78	-5.213	0.3962	1	0.2379
Xe-133	0	0	0.003434	11.26	0.003569	0.8714	0.02258
Xe-134	0	0	47.23	-2.982	40.72	1	2.256
Xe-135	0	-0.0001477	0.01857	-0.0655	0.00009651	0.9995	0.003473
Xe-136	0	0.15	63.48	2.188	12.2	1	1.32
Kr-82	0	0.0004344	-5.311E-05	0.0004301	8.664E-07	1	0.00038
Kr-83	0	-0.01415	1.892	0.08949	0.01173	1	0.04093
Kr-84	0	0	3.479	0.4714	0.2076	1	0.1611
Kr-85	0	-0.005164	0.9572	0.09066	0.01097	1	0.03703
Kr-86	0	-0.02978	7.515	0.799	0.6271	1	0.28
I-127	-0.0001673	0.01877	0.7931	-0.3614	0.1547	1	0.1487
I-129	-0.0004874	0.04896	3.159	-0.5321	0.563	1	0.2836
I-130	1.346E-08	2.359E-06	0.0001224	0.00002983	1.069E-09	1	0.00001335
I-131	0	0	0.02174	7.592	0.008056	0.9828	0.03173
I-135	-2.615E-07	0.00002467	-0.0005749	0.5578	3.497E-06	0.8855	0.0005914

Table 1 summarizes the values of the constants of the inferred equations for the concentration of each isotope, in addition to the statistical indicators, on the basis of which (best) solution is chosen. As can be seen from “Table 1,” the second-order polynomial regression analysis was the dominant solution for a large number of isotopes (8 out of 19 cases), and close to that the third-order polynomial regression analysis (7 out of 19 cases), while we find that the first order polynomial regression or linear regression were representative in only 4 cases.

Before adopting the inferred equations for each case, the statistical determinants were calculated to determine the effectiveness of the model or solution, which are RMSE and R^2^. As seems clear from the results in “Table 1,” all RMSE values are very close to zero, which indicates how near the predicted equations are. On the other hand, we found that all R^2^ values are incredibly close to 1, and even (15 out of 19) of them are actually equal to 1, and the remaining four values range from 0.8714 to 0.9995. These values for R^2^ show that the inferred equations are valid for all data points. These statistical indicators ensured for us the quality of the measurement of both the correlation and the accuracy between the inferred equations and the data points.

Concentration (g/tHM) versus burnup (GWD/tHM) for all xenon and krypton isotopes resulting from fuel burnup from 0 to 25 GWD/tHM has been plotted along with the inferred equations as illustrated by “S3 and S5 Figs,” respectively.

The extracted equations have been generalized for all cases to include fuel burnup from 25 to 51 GWD/tHM with a comparison between them and the values taken from the MCNP outputs for the same fuel burnup range as shown in “S4 and S6 Figs,” respectively. This comparison was made using Eq (1). S7 and S8 Figs show the discrepancies for xenon and krypton isotopes, respectively. The discrepancy % is an indication of the accuracy of the regression or the magnitude of error between the data points and the corresponding predicted ones. Discrepancies for xenon and krypton were found to range from -1.5% to 3.5% and from -0.25% to 1.5%, respectively. These small values of discrepancy (3.5% as maximum) demonstrate the strong matching of the inferred solutions to the point values of the fission product concentrations of xenon and krypton isotopes taken from the MCNP.

## Conclusion

In this research, a simple mathematical method (regression) was used, which most researchers in the field can deal with and for which there are a large number of programs specially prepared for such calculations. In particular, we used MATLAB and its built-in Curve Fitting Toolbox functionality.

MATLAB was used to infer the relationship between the predictor (the Burnup of nuclear fuel) and the response variables (the concentrations of fission products of both xenon and krypton isotopes). It was assumed that linear regression fits the data points taken from the output of the MCNP program, and this assumption was proven correct after using the most common type of linear regression, a polynomial least squares fit. “Table 1” shows the accuracy of the inferred equations for all nineteen cases, as all the statistical indicators of the equations are close to ideal. Nevertheless, this research does not diminish the importance of using specialized codes of nuclear fuel and programs such as MCNP, Origin and others. Rather, it gives a strong indication of the importance of integration between them. This simple method that relied on data taken from these programs for only part of fuel burnup (from 0 to 25 GWd/tHM) yielded results obtained for the rest of the fuel cycle (from 25 to 51 GWd/tHM) were unexpectedly good.

Based on this method, researchers can predict the concentration of any component of the fuel, whether this fuel is still in use inside the reactor or is spent. Not only the fission products whose concentration can be predicted, but even the actinides already present in the fuel or even formed as a result of nuclear reactions or decay within the fuel. In this research, we presented an example of predicting gaseous concentrations of xenon and krypton isotopes, which are the most important gaseous fission products. Researchers can study gaseous bubbles that may cause problems in nuclear fuel, and this method can be useful in predicting any isotope or isotopes of interest in nuclear fuel research such as predicting the amount of neutron poisons that disrupt the operation of reactors after they are shut down. Also, this method may be very useful when studying fuel recycling or storage or achieving high fuel burnup rates for the current fuel inside the reactors (for example, 100 GWd/tHM burnup). The use of this method opens countless areas in predicting the concentrations of different isotopes in the nuclear fuel cycle, which enhance nuclear safety.

## Supporting information

S1 Fig MCNP model of a 17x17 pins Westinghouse pressurized water reactor fuel assembly. (TIF)

S2 Fig Flowchart of simulation and regression processes. (TIF)

S3 Fig Regression (Lines) of Xenon isotopes concentrations using burnup data from MCNP (data points). (TIF)

S4 Fig Comparison between predicted equations from regression (Lines) with MCNP data (data points) of Xenon isotopes concentrations during burnup period of (25 to 51 GWd/tHM). (TIF)

S5 Fig Regression (Lines) of krypton isotopes concentrations using burnup data from MCNP (data points). (TIF)

S6 Fig Comparison between predicted equations from regression (Lines) with MCNP data (data points) of krypton isotopes concentrations during burnup period of (25 to 51 GWd/tHM). (TIF)

S7 Fig Discrepancies % between the predicted equations and MCNP data points for Xenon isotopes concentrations. (TIF)

S8 Fig Discrepancies % between the predicted equations and MCNP data points for Krypton isotopes concentrations. (TIF)

S1 Data (XLSX)

S1 File MCNP input file. (TXT)

S2 File Poly-regression code. (M)
