# Supplementary figures and images for: Accurate prediction of gaseous isotopes Xe and Kr concentrations from the burnup of nuclear fuel using simple regression algorithm
Source: PLoS One. 2023 Jul 13;18(7):e0288329. doi: 10.1371/journal.pone.0288329 (PMC10343056; doi:10.1371/journal.pone.0288329)

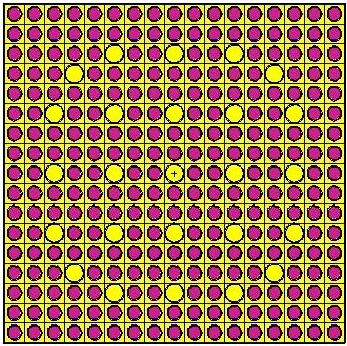

Supplement: S1 Fig — (TIF) [file pone.0288329.s001.tif]

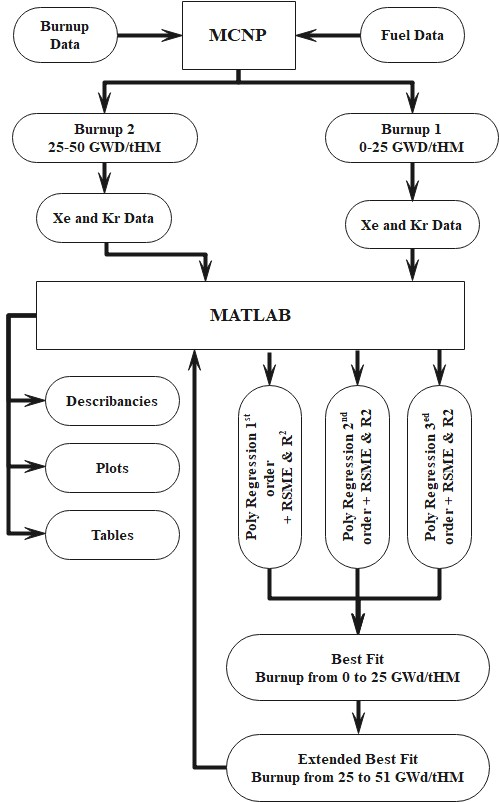

Supplement: S2 Fig — (TIF) [file pone.0288329.s002.tif]

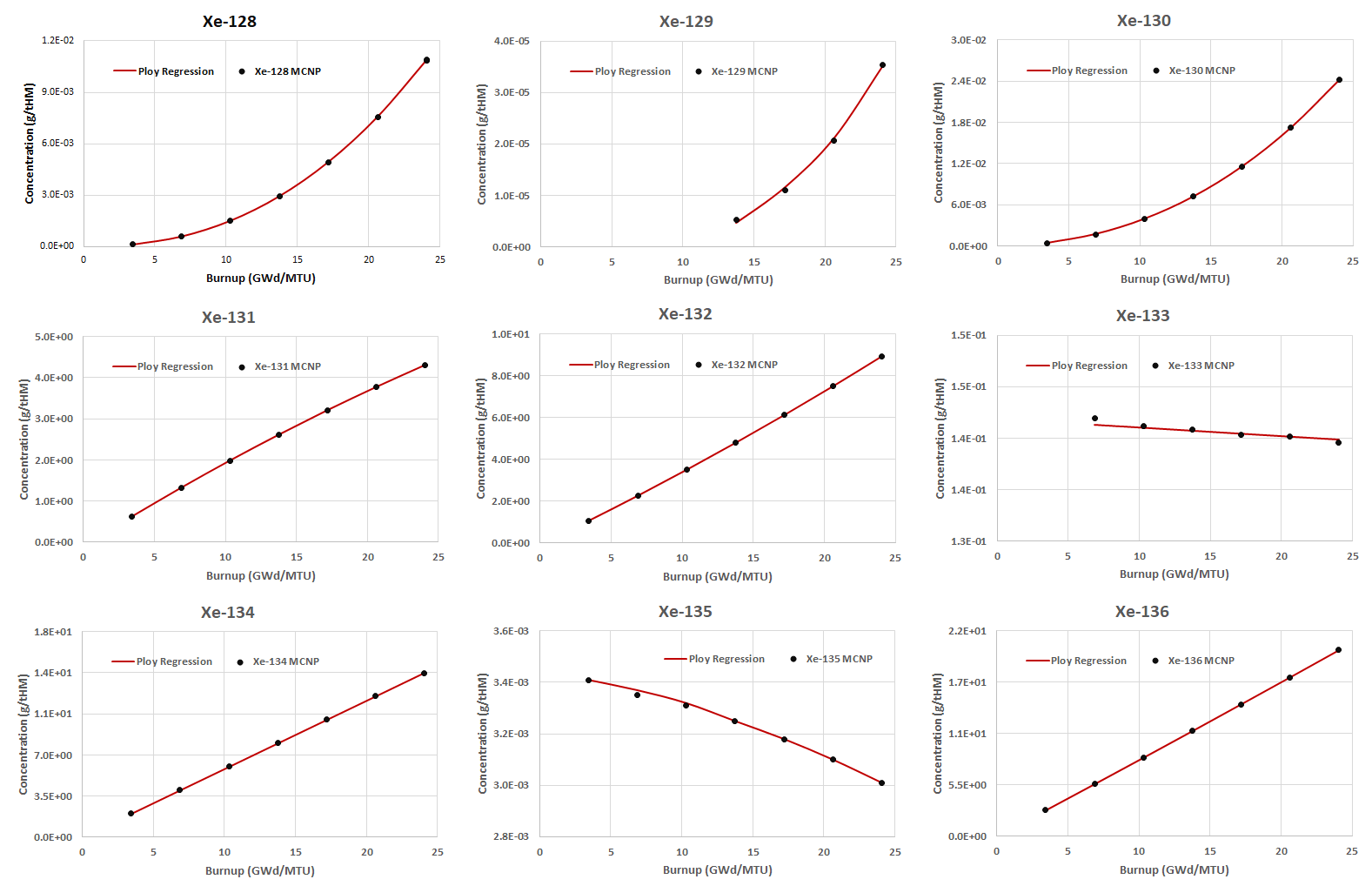

Supplement: S3 Fig — (TIF) [file pone.0288329.s003.tif]

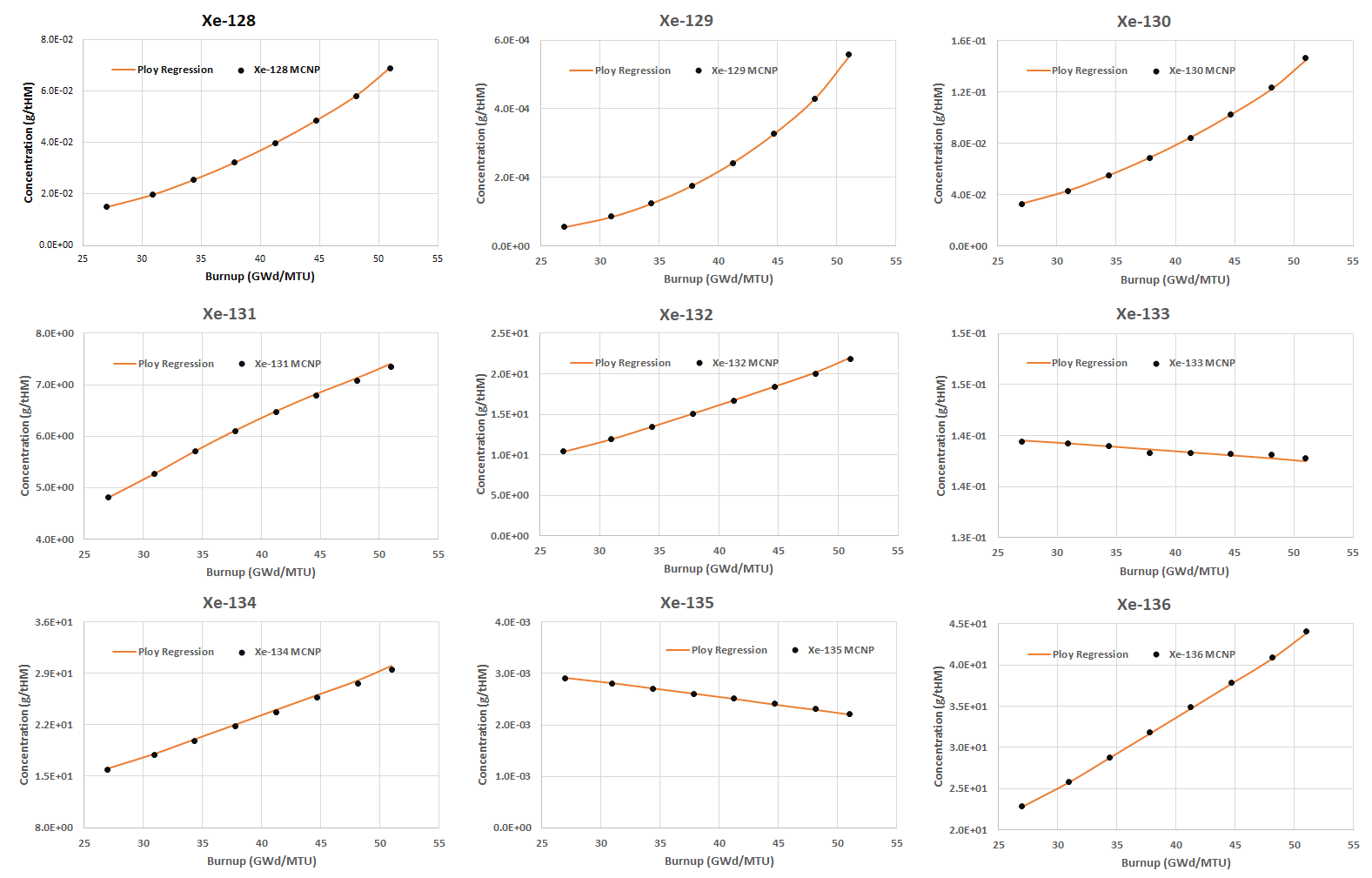

Supplement: S4 Fig — (TIF) [file pone.0288329.s004.tif]

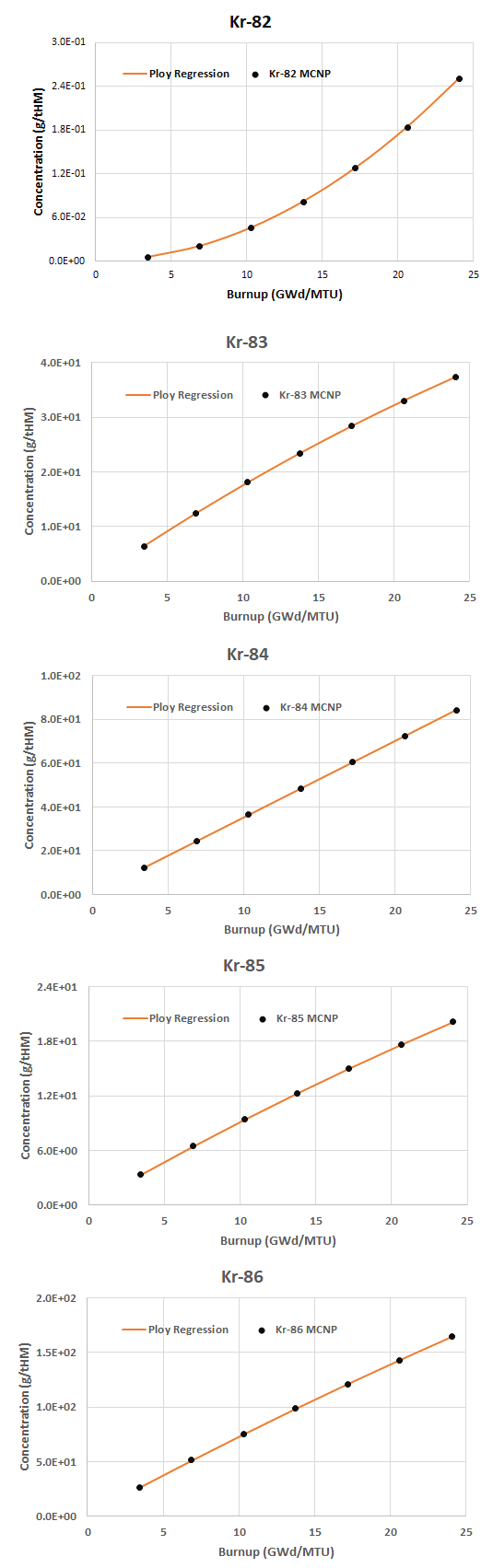

Supplement: S5 Fig — (TIF) [file pone.0288329.s005.tif]

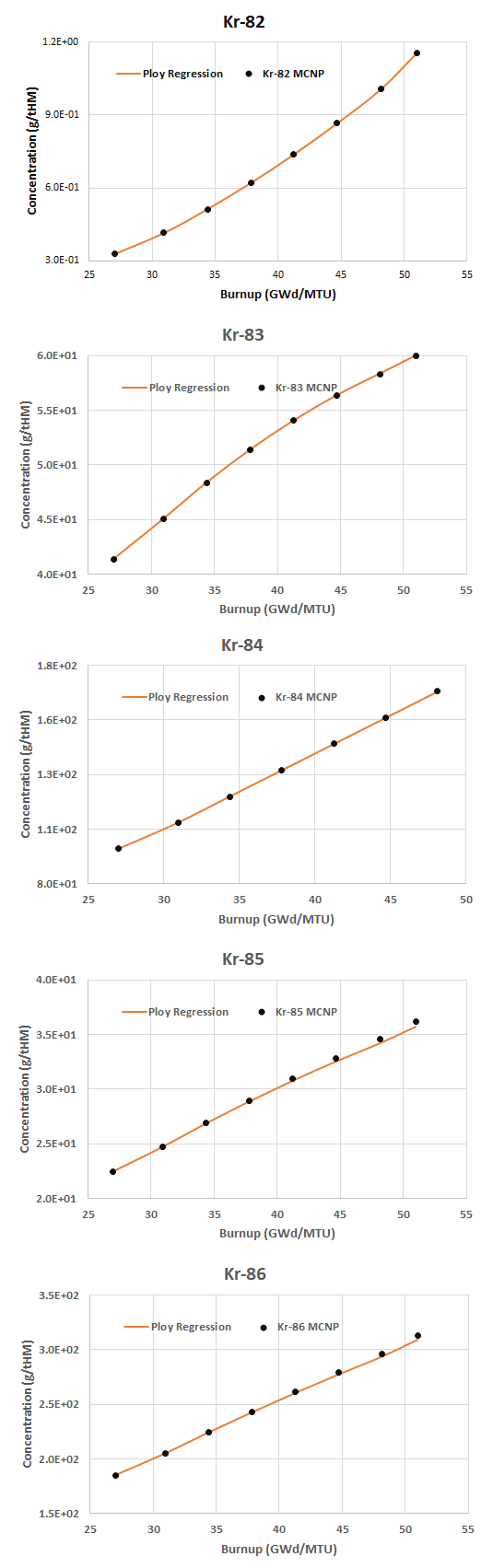

Supplement: S6 Fig — (TIF) [file pone.0288329.s006.tif]

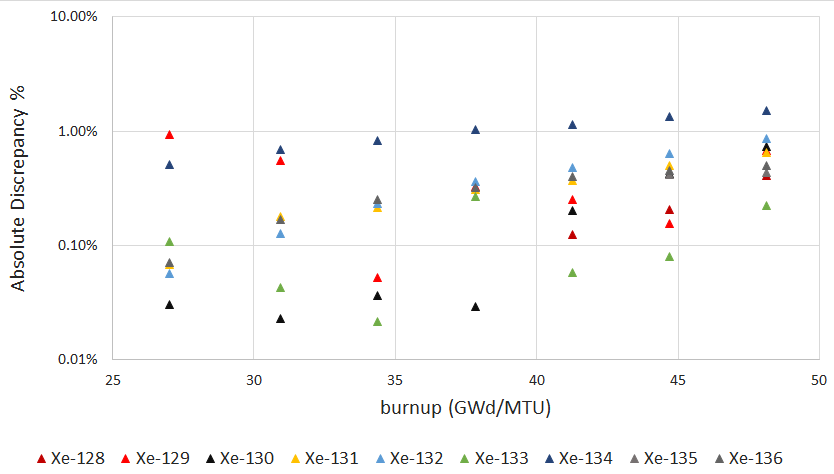

Supplement: S7 Fig — (TIF) [file pone.0288329.s007.tif]

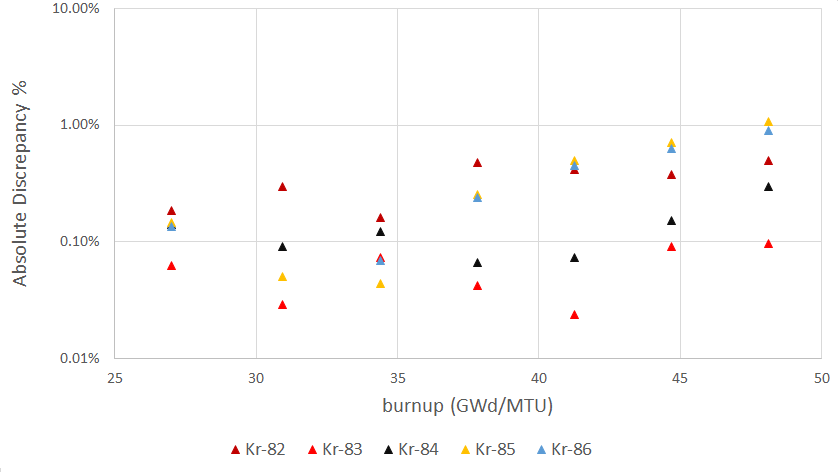

Supplement: S8 Fig — (TIF) [file pone.0288329.s008.tif]
